# Relationship between Knee Muscle Strength and Fat/Muscle Mass in Elderly Women with Knee Osteoarthritis Based on Dual-Energy X-Ray Absorptiometry

**DOI:** 10.3390/ijerph17020573

**Published:** 2020-01-16

**Authors:** Xini Zhang, Xiaoyu Pan, Liqin Deng, Weijie Fu

**Affiliations:** 1 School of Kinesiology, Shanghai University of Sport, Shanghai 200438, China; zhangxini1129@163.com (X.Z.); xiaoyutiankong134@163.com (X.P.); liqind22@gmail.com (L.D.); 2 Key Laboratory of Exercise and Health Sciences of Ministry of Education, Shanghai University of Sport, Shanghai 200438, China

**Keywords:** muscle strength, fat/muscle mass, knee osteoarthritis, dual-energy X-ray absorptiometry

## Abstract

*Purpose*: This study aimed to examine the characteristics and correlation of knee muscle strength and body composition (fat and muscle mass) among elderly women aged 60–70 years with knee osteoarthritis. The present study hypothesized that the muscle mass and the peak torques of the knee joints were considerably low in the knee osteoarthritis (KOA) group. *Methods*: A total of 47 elderly women aged 60–70 years were recruited from Yangpu District in Shanghai and assigned to the knee osteoarthritis (*n* = 25, KOA) or healthy control group (*n* = 22, CON). The knee extension/flexion isokinetic strength measurements were conducted on an isokinetic dynamometer at angular velocities of 90°/s. Dual-energy X-ray absorptiometry was used to measure the body composition (fat and muscle mass in the whole body and lower limbs). The independent sample t-test was employed to determine the effects of knee osteoarthritis on each variable, and the Pearson correlation analysis was used to investigate the correlation between the body composition and knee muscle strength. *Results*: Compared with the CON, the KOA exhibited the following: (1) Lower absolute peak knee extension torque (66.02 ± 10.57 vs. 56.61 ± 14.69 Nm), relative peak knee extension (1.11 ± 0.19 vs. 0.89 ± 0.26 Nm/kg), and flexion torque (0.62 ± 0.15 vs. 0.54 ± 0.16 Nm/kg, *p* < 0.05); (2) greater relative peak torque ratio of the knee extension and flexion (0.55 ± 0.08 vs. 0.62 ± 0.15, *p* < 0.05); and (3) lower total body muscle mass percentage (63.24% ± 4.50% vs. 59.36% ± 3.94%), particularly in the lower limbs (19.96% ± 1.51% vs. 18.47% ± 1.49%, *p* < 0.05). Furthermore, the total body fat mass percentage was negatively associated with the relative peak knee extension and flexion torque regardless of the group (*p* < 0.05). The total body muscle mass percentage was positively associated with the relative peak knee extension torque in the two groups and the relative peak knee flexion torque in the CON (*p* < 0.05). *Conclusion*: For elderly women with knee osteoarthritis, knee muscle strength decreases significantly, especially for the extensor strength. Moreover, compared with fat mass, the index of muscle mass is more sensitive in detecting the decrease in knee joint torque. Therefore, rather than weight loss alone, the quadriceps muscle and the rear-thigh muscles, which maintain the stability of knee joints during rehabilitation training, should be strengthened emphatically to improve muscle mass.

## 1. Introduction

Osteoarthritis is a common arthritis disease amongst the elderly. Patients with knee osteoarthritis (KOA), one of the main causes of disability, account for 40% of the elderly with osteoarthritis over 65 years old [1]. Patients with KOA suffer from joint pain, joint stiffness, and loss of muscle strength of the lower limbs, resulting in declines in functional status and limitation of limb movement [2]. Previous studies have shown that, compared with those of healthy individuals, the quadriceps femoris and rear-thigh muscles of patients with KOA are significantly damaged [3]. At present, the two main factors known to affect muscle strength are the size of the muscle cross-sectional area and nerve control of the activated muscle [4]. Many studies have shown that the cross-sectional area and muscle strength of the quadriceps femoris of patients with KOA are smaller than those of cases without the condition [5,6]. In addition, epidemiological studies have shown muscle composition and obesity to be associated with many chronic diseases, including osteoarthritis [7,8,9]. As aging progresses, loss of muscle mass is often accompanied by an increase in fat, a contributing factor in the growth of elderly obesity [10]. It is believed to decrease muscle strength, mechanical stress, and inflammation, all of which can lead to KOA.

Many experts and scholars have proposed strengthening of the quadriceps femoris muscle and reducing weight to prevent or recover from KOA [11]. Increasing the strength of the quadriceps femoris muscle may reduce the wear of the lateral patella–femoral articular cartilage and protect the articular cartilage, thereby reducing the risk of KOA [12]. Weight loss can reduce the burden of the lower limbs and ease the pain of patients [13]. However, quadricep strengthening exercises do not decrease the incidence of KOA [14]. This finding is attributed to the focus on only the mechanical properties of the quadriceps femoris of patients with KOA but not on the muscle strength of the rear-thigh muscles, which maintain the stability of knee joints, as well as the proportions of muscle and fat mass [15]. Notably, the atrophy of quadriceps femoris is a risk factor and a consequence of the inactivity and disuse of the “diseased” limb [16]. Based on previous studies, the present study focuses on the strength of the extensor and flexion muscles simultaneously to enable the development of the appropriate therapies [17].

Dual-energy X-ray absorptiometry (DXA) is often used to measure the bone mineral density of the human body [18,19]. This method is widely used on account of its low cost, simple operation, and reliable results. A recent systematic review showed that DXA could be regarded as a new gold standard for measuring the body composition because it is highly correlated with nuclear magnetic resonance and computed X-ray tomography [20]. Up until now, DXA has been used to measure the bone mineral density and the body composition of athletes, thus providing a suitable reference for the training and diet plan [20]. However, few studies have applied DXA to measure the body composition of patients with KOA. Therefore, the present study aimed to determine muscle and fat masses in KOA on the basis of DXA, and their relationship with muscle strength of the lower limbs, to investigate the pathological mechanism of KOA.

The main purpose of this study was to explore the characteristics of knee joint muscle strength and body composition (fat mass and muscle mass) and their correlations in elderly female patients with KOA. In this work, patients with KOA were compared with age- and gender-matched healthy controls (CON). The results of this work may help further improve rehabilitation programs for KOA amongst the elderly to prevent and reduce the risk of injury. The following were the experimental hypotheses: (1) Fat mass was significantly higher whilst muscle mass was significantly lower in the KOA group than in the CON group, (2) the absolute and relative peak torques of knee–joint flexion and extension in the KOA group were significantly lower than those in the CON group, and (3) the fat mass and flexor/extensor strength of the knee joint were significantly negatively correlated whereas the muscle mass and flexor/extensor strength of the knee joint were significantly positively correlated amongst individuals with or without KOA.

## 2. Materials and Methods

### 2.1. Participants

The experimental information was distributed to the elderly through each community committee from Yangpu District in Shanghai. No remuneration was offered for participation. A priori power analysis was conducted and indicated a total sample size of 42 with α error of 0.05 and a power of 0.8 (G*Power v3.9.1.4 software, Institute of Experimental Psychology, Kiel, Germany). Considering the attrition rate, the present study has recruited 47 women aged 60–70 years, including 25 patients in the KOA group and 22 participants in the CON group. All subjects gave their informed consent for inclusion before they participated in the study. The study was conducted in accordance with the Declaration of Helsinki, and the protocol was approved by the Ethics Committee of Shanghai University of Sport (No. 2013001).

**Figure 1:**
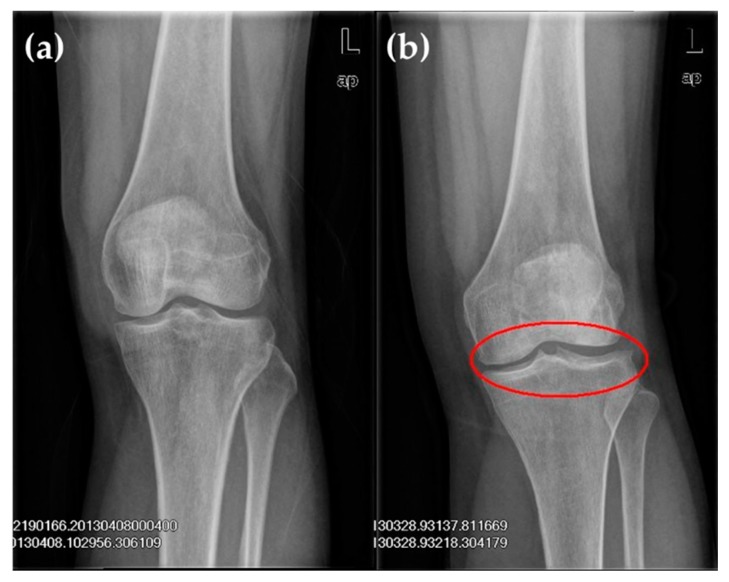
X-ray film of left knee joint: (**a**) The normal knee showed no obvious abnormal change in the sclerotin of bones in the joint, a well-proportioned joint gap and no obvious swelling of surrounding soft tissue; (**b**) The knee osteoarthritis (KOA) knee showed protruded left tibial condyles; labial hyperplasia in the external condyle of the tibia, margin of the lateral condyle of the femur and posterior margin of the patella; and a well-proportioned joint gap.

The following were the inclusion criteria for the KOA group: (1) Clinical diagnosis standard for knee arthritis by the American Rheumatology Association in 1986 [21]; and (2) imaging features (Figure 1). Patients with KOA were graded on the basis of the Kellgren–Lawrence grading criteria (K–L grading) (Table 1) [22]. Anterior, posterior, and lateral radiographies of the knee joint were commonly used to determine the X-ray projection positions of the joint. During anterior and posterior radiographies, patients lied on their backs, and the X-ray was inclined 5° to the cephalic side [23,24]. During lateral radiography, patients bent their knee joint at a 20°–35° angle, and the joint gap amongst internal, external tibiofemoral joints and patellofemoral joints were clearly observed.

The following were the exclusion criteria for the KOA group: (1) Inability to walk or walking distance of less than 7.6 m; (2) the knee joint was undergoing drug treatment or intra-articular injection or underwent even bilateral knee joint replacement; (3) uncontrollable angina pectoris induced by exercise, uncontrolled hypertension, acute or chronic renal failure, dyspnoea in the resting state, and terminal disease occurring within 3 months; and (4) a score of less than 27 points on the simple mental state checklist [12].

### 2.2. Experimental Process and Data Acquisition

Laboratory personnel were organized to carry out a pre-experiment prior to the formal experiment. All experimental personnel were familiar with the experimental procedure, standard operation, and scale testing; they also prepared the experimental supplies and arranged the experimental site. The participants were divided into groups, informed of the experimental procedure and asked to sign informed consent forms. Measurements were made on the basis of the Western Ontario and McMaster Universities Index (WOMAC), which evaluates pain, stiffness, and physical dysfunction by using a 0–10 cm visual simulation scoring method. In this case, 0 cm means no pain, joint stiffness or physical dysfunction, whereas 10 cm represents severe pain, joint stiffness, or severe limitation of physical function. The scale comprises 24 questions: 5 questions on pain evaluation, 2 questions on joint stiffness, and 17 questions on physical function [19]. The WOMAC scale has been widely used to evaluate hip and knee joint dysfunction and is a standard questionnaire for evaluating pain, joint stiffness, and dysfunction amongst patients with osteoarthritis [20]. A high score on the WOMAC index indicates severe OA. The evaluation standard is mild < 80, moderate 80–120, and severe > 120.

The body compositions of participants were determined using DXA (Lunar Prodigy, US GE Company, Boston, USA). Participants were required to wear a uniform bathrobe, and all articles containing metal components (metal accessories and bras) were removed before testing. Then, participants were required to lie on their backs at the centre of the test platform, with their palms facing downwards on both sides of the body in parallel. They were scanned from head to feet for approximately 5 min. Built-in analytical software was used to acquire and analyze the percentages of total body muscle mass (TMM, %), fat mass (TFM, %), muscle mass of the lower limbs (LMM, %), and fat mass of the lower limbs (LFM, %).

A multi-joint dynamometer (Con-trex multi-joint model, CMV AG, Dübendorf, Switzerland) was used to measure the flexor and extensor torque of the knee joint of the unilateral symptomatic leg in the KOA group and the dominant leg in the CON group. All participants were required to assume the sitting position, and the centripetal test of isokinetic strength of the knee joint was conducted. The participants were seated, with a fist distance from knee joint to the edge of the seat. The torso and thighs were fixed with belts, and the non-tested legs were fixed by the instrument. The angular velocity of the constant-speed power head instrument was 90°/s (centripetal/centripetal). An angular velocity of less than or equal to 60°/s is generally defined as strength testing according to a standardized protocol [21]. However, an angular velocity of 60°/s increases the friction of the joint and even aggravates the symptoms of the injured person. All participants in the KOA group were elderly patients with KOA; thus, the angular velocity for the test was increased to 90°/s to ease the re-abrasion of the knee joint amongst patients with KOA. Prior to formal testing, the test leg was subjected to de-gravitation correction, and the maximum contraction test was conducted. When the participants had become familiar with the isokinetic muscle strength test, they participated in three rounds of the repeated maximum contraction isokinetic centripetal test. The absolute peak torque (aPT, Nm), relative peak torque (rPT, Nm/kg) of the knee flexion and extension, and relative peak torque ratio (H/Q) were collected and calculated.

### 2.3. Statistics

All parameters are expressed as mean ± standard deviation. The Shapiro–Wilk *W* test was used to verify the normality of data distribution. The independent sample t-test (SPSS 21.0, Armonk, New York, USA) was used to compare the age, height, weight, aPT, rPT, H/Q, TMM, TFM, LMM, and LFM of the two groups. Pearson correlation was used to analyze the correlation between body composition (TFM, TMM, LFM and LMM) and knee joint muscle force (extension rPT, flexion rPT) of the two groups. The correlation coefficient was set to r, and the significance level α was set to 0.05.

## 3. Results

### 3.1. Basic Information and Clinical Diagnosis of Paricipants

The weight of patients in the KOA group was significantly higher than that of participants in the CON group (*p* < 0.05). In the WOMAC osteoarthritis index scale, the total score of the KOA group was less than 80 points, diagnosed with mild KOA (Table 2).

### 3.2. Body Composition Based on DXA and Knee Joint Muscle Strength

**Figure 2:**
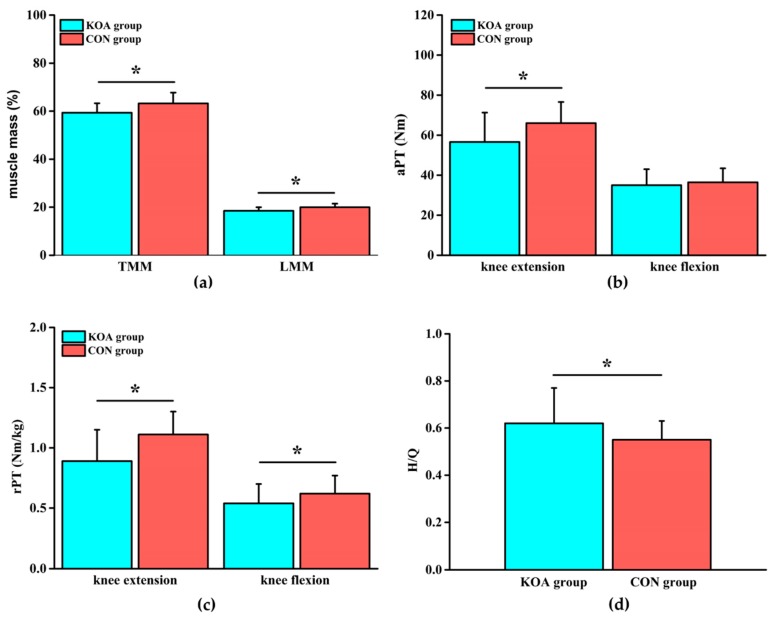
(**a**) Comparison of the muscle mass (total muscle mass (TMM) and lower limbs muscle mass (LMM)) between the KOA and control group (CON) groups; (**b**) comparison of the absolute peak torque (aPT) of the knee joint between the KOA and CON groups; (**c**) comparison of the relative peak torque (rPT) of the knee joint between the KOA and CON groups; and (**d**) comparison of the relative peak torque ratio (H/Q) of knee flexion and extension between the KOA and CON groups. * means *p* < 0.05.

Compared with those of the CON group, TMM in the KOA group was reduced by 6.5% and LMM was reduced by 8.1% (Figure 2a, *p* < 0.05). The differences in TFM and LFM between the two groups were not significant (Table 3). Compared with those of the CON group, the extension aPT in the KOA group was lower by 16.6%, the extension rPT was lower by 24.7%, and the flexion rPT was lower by 14.8% (Figure 2b, c, *p* < 0.05). There was no significant difference in flexion aPT between the two groups (Table 4). The difference in H/Q was significantly increased in the KOA group, compared with that in the CON group (Figure 2d, *p* < 0.05).

### 3.3. Correlation between Knee Joint Muscle Strength and Body Composition

In the KOA group, rPT was negatively correlated with TFM and positively correlated with LMM in both knee extension and knee flexion. Flexion rPT was also positively correlated with TMM (Table 4, *p* < 0.05). In the CON group, rPT was negatively correlated with TFM and LFM and positively correlated with TMM and LMM in both knee extension and knee flexion (Table 4, *p* < 0.05). 

## 4. Discussion

Differences in the knee joint muscle force and body composition (fat mass and muscle mass) between the KOA and CON groups and their correlations were compared based on DXA and a Con-trex isokinetic dynamometer. The weight of the KOA group was significantly higher than that of the CON group. For body composition, TMM and LMM in the KOA were significantly lower than those in the CON group, whilst the differences in TFM and LFM between two groups were not significant; these results partly supported Hypothesis 1. Knee joint muscle force, extension aPT and rPT, and flexion rPT were significantly reduced whereas H/Q was significantly increased in KOA; these findings supported Hypothesis 2. Finally, extension and flexion rPT in the KOA group were negatively correlated with TFM and positively correlated with LMM. Flexion rPT was also positively correlated with TMM. In the CON group, extension and flexion rPT are negatively correlated with TFM and LFM and positively correlated with TMM and LMM; these results supported Hypothesis 3.

The body composition measured by DXA reveals that TMM and LMM were decreased significantly in the KOA group; in particular, the decrease in LMM was larger than that in TMM. Thus, the total body muscle mass of patients with KOA, especially lower limb muscle mass, was lost. Karlsson et al. found that the significance of the lead body weight of elderly women with KOA was smaller than that of the CON group [25]. Abbate et al. also found that, although the lean body weight of the KOA group was significantly greater than that of the CON group, the percentage of this parameter on the former was significantly lower than that of the latter [26]. This finding was consistent with the results of the present study. Muscle mass is replaced by fat as the human body ages [25]. In the present study, all of the participants were elderly. This detail may explain the non-significant differences in TFM and LFM between the two groups. However, the body weight of patients with KOA was significantly higher than that of the CON group. Obesity was believed to increase the risk of KOA [25,26,27]. The risk of KOA increased by 36% as body weight increased by 5 kg [27]. Cadaver studies have shown that the prevalence of KOA amongst obese patients is higher than that amongst individuals with normal weight [28]. The musculoskeletal system bore the burden caused excess weight as the body weight increased, thereby increasing pain amongst patients with KOA; as pain becomes more severe, patients with obesity may refuse to participate in regular daily activities and gain weight faster [13], and this factor led to a vicious circle. Therefore, increasing muscle strength and controlling weight during the rehabilitation training of KOA for the elderly should be emphasized.

The muscle force around the knee joint played an important role in maintaining the stability of the knee joint. It could prevent the occurrence or further development of KOA [29]. By contrast, loss of muscle mass led to a decrease in muscle force, which may cause KOA [10]. This experimental result showed that knee extension aPT and rPT, as well as knee flexion rPT, significantly reduced in the KOA group. The extensor group of the knee joint was mainly the quadriceps femoris, and the flexor group was mainly the hamstring. Therefore, knee extension and flexion torque could reflect the muscle strength level of the quadriceps femoris and hamstring. Previous studies have shown that a decline in quadriceps femoris muscle strength is common amongst patients with KOA. Hassan et al. found that, compared with that of the control group, the quadriceps femoris muscle strength of patients with KOA was weaker, leading to increased postural instability [30]. Messier et al. also reported that the isokinetic muscle strength of the quadriceps femoris of patients with KOA decreased by 22–36% compared with that of healthy controls [31], which was similar to the decrease of 19.8% in the extensor muscle strength in the present study [32]. The muscle strength of the lower limbs provided stability to the knee joint; weak quadriceps femoris muscle strength was usually accompanied by a higher load rate and resulted in a narrow joint space, which may cause KOA [33]. As the extensor muscle strength of the knee joint of patients with KOA was commonly decreased, improving the extensor muscle strength of the knee joint had become an important basis of rehabilitation training. Most of previous studies focused on changes in quadriceps femoris muscle strength amongst patients with KOA, and few have aimed to analyze the flexor muscle group of the knee joint, which jointly maintain its stability. Wildcy et al. proposed that decreased hamstring muscle strength resulted in misalignment of the frontal plane of the knee joint in patients with KOA; this misalignment caused the control ability of the frontal plane of the knee joint to decrease, thereby increasing the load on the anterior cruciate ligament and the risk of injury [34]. 

As an index reflecting the stability of the knee joint, H/Q was used to evaluate the relationship between flexors and extensors and the balance of joint muscle strength. It was crucial to maintain normal index in efforts to prevent the elderly from falling [35]. The H/Q of healthy elderly women was from 40% to 55% [36], which was higher in KOA group than the normal range. Patsika et al. found that the ratio of the flexor and extensor muscles amongst female patients with KOA was significantly higher than that amongst healthy controls, which was mainly due to a decrease in quadriceps femoris muscle strength [36]. Tan et al. found that the ratios of the flexor and extensor muscles between patients with KOA and healthy individuals were not significantly different. This finding was attributed to the equal decrease in strength of the quadriceps femoris muscle and hamstring muscle [37]. In the present study, H/Q in the KOA group was significantly higher than that in the CON group, and the strengths of the extensor and flexor muscles decreased by 24.7% and 14.8%, respectively. Therefore, although the muscle strength of the extensor and flexor decreased, the decrease in strength of the extensor muscle was greater than that of the flexor muscle, thus suggesting that the muscle strength of the flexor and extensor in the knee joints of patients with KOA decreased asynchronously. Given the significant decrease in strength of the extensor muscle amongst patients with KOA, a decrease in stability may be expected. The rehabilitation training plans for patients with KOA should emphasize the extensor muscle strength of the knee joint. The hamstring muscle should also be strengthened to maintain balance between muscle strength and knee joint stability.

In the present study, a Pearson correlation analysis showed that TFM was significantly negatively correlated with the muscle strength of lower limbs in the presence or absence of KOA; thus, the muscle strength of the lower limbs decreased with increasing TFM. This finding was similar to that of Newman et al. A high body fat rate was related to the weak muscle strength of lower limbs, and a high body fat rate could accelerate the process of KOA [38]. Henriksen found that weight loss was accompanied by an increase in muscle strength amongst 159 elderly patients with KOA on a 16-week low-calorie diet, and resulted in improvements in physical function and knee pain [39]. Therefore, the body weight, fat mass, and muscle strength of the lower limbs affected one another. The mechanical mechanism of obesity played an important role in the muscle strength of lower limbs [40]. When the muscle strength of the lower limbs weakened, the muscles were unable to absorb the excessive stress caused by weight gain, thereby increasing the risk of KOA [6]. However, not all studies have found a correlation between adipose tissue and muscle strength of the lower limbs. Segal found no significant differences in muscle strength between different body mass index populations and patients with KOA. In addition, intramuscular fat and peak torque of quadriceps femoris were not correlated [41]. Considering that participants in the previous study were elderly people aged 50–59 years whilst the participants in the present study are aged 60–70 years, we believed that different ages may lead to different results. 

In the present study, no correlations in extension rPT between TMM and KOA were found. TMM and LMM were significantly positive correlated with knee joint extension and flexion rPT in the KOA and CON groups. TMM and LMM in the KOA group were significantly lower than those in the CON group. Therefore, loss of muscle mass amongst patients with KOA had a considerable effect on the decrease in muscle strength of the lower limbs. Sarcopenia referred to the decrease in muscle mass with age and is the main reason for the decline in muscle strength of the elderly. Newman believed that the low muscle strength of the elderly was mainly due to their low muscle mass, which verified the relationship between muscle mass and KOA [38]. Slemenda et al. found that decreases in muscle strength were highly correlated with muscle mass, by monitoring the physical composition of the bodies of female patients with KOA via DXA [42]. This study showed that fat mass and muscle mass were correlated with decreased muscle strength in patients with KOA. Specially, the knee joint muscle strength, which was related to the muscle strength of the lower limbs, showed a corresponding decrease in LMM with muscle mass but no significant correlation with LFM. At present, nearly all clinical guidelines recommend the use of non-drug therapies for rehabilitation, amongst which exercise therapy play a major role [11,43]. Therefore, when choosing exercise methods, patients with KOA should not only consider the effect of weight loss but also that of muscle mass [44]. According to the guidelines of the Osteoarthritis Research Society [45], preliminary results from our previous studies have shown that Tai Chi was an excellent exercise therapy. Tai Chi significantly reduces knee pain and stiffness in patients with KOA and effectively controls weight after six months of training [46,47]. As such, resistance exercise (e.g., Tai Chi) may be applied to patients with KOA.

The present study had several limitations. Although all data normalized by body weight were not affected by non-matched weight in two groups, matching the weights of the two groups may be better. Given the small sample size, comparative analysis by age was not possible. In future research, the age range can be expanded using a more heterogeneous group of patients with KOA by comparing index differences amongst different ages. The sample size can also be increased to discuss the classification of patients in KOA. In the isokinetic muscle force test of the knee joint in this study, only the isokinetic flexor and extensor torques of the knee joint at 90°/s angular velocity were tested. In the future, the angular velocity setting should be examined and bilateral legs should be tested to explore the correlation between knee joint muscle strength and body composition on the basis of multiple indices. More parameters (e.g., kcal for diet and physical activity level) can be considered in the future when conducting a correlation analysis.

## 5. Conclusions

The flexor and extensor muscle strength of the knee joint of elderly female patients with KOA significantly decreased, particularly the latter. Therefore, rehabilitation training should focus on exercising the extensor muscle to build its strength. Moreover, improvement of the flexor muscle strength should be taken into account to maintain the balance between muscle strength and stability of the knee joint. Muscle mass is a more important index than fat mass in reflecting the decline of knee joint muscle strength amongst elderly women. Therefore, weight loss and improvement of muscle mass should be highlighted during rehabilitation training.

## Figures and Tables

**Table 1:** Kellgren–Lawrence grading criteria.

Grade	X-ray Imaging Features
0	Normal
I	The joint gap is suspected to be narrowed; there may be osteophyma
II	Obvious osteophyma; the joint gap is suspected to be narrowed
III	Moderate osteophyma; the joint gap is obviously narrowed with sclerotic change
IV	Massive osteophyma; the joint gap is obviously narrowed with severe sclerotic lesions and obvious deformities

**Table 2:** Basic information and clinical diagnosis of participants.

Basic Information/Clinical Diagnosis	KOA Group (*n* = 25)	CON Group (*n* = 22)
Age (years)	64.42 ± 2.95	62.25 ± 2.38
Height (cm)	155.92 ± 5.90	157.07 ± 4.11
Weight (kg)	62.80 ± 9.30 *	55.79 ± 5.2
K–L grading		
Grade 0	0	-
Grade I	6	-
Grade II	14	-
Grade III	5	-
Grade IV	0	-
WOMAC scoring		
Pain (0–50 points)	10.8	-
Stiffness (0–20 points)	3.96	-
Body function (0–170 points)	35.32	-
Total score (0–240 points)	50.08	-

Note: KOA group is knee osteoarthritis group, CON group is control group, K–L grading is Kellgren–Lawrence grading criteria, and WOMAC is Western Ontario and McMaster Universities Index. * *p* < 0.05.

**Table 3:** Comparison of knee joint peak torque and body composition of participants between the KOA and CON groups (mean ± SD).

Body Composition/Peak Torque	KOA Group (*n* = 25)	CON Group (*n* = 22)
Muscle mass		
TMM (%)	59.36 ± 3.94 *	63.24 ± 4.50
LMM (%)	18.47 ± 1.49 *	19.96 ± 1.51
Fat mass		
TFM (%)	35.65 ± 4.18	33.00 ± 6.93
LFM (%)	9.29 ± 1.87	9.43 ± 2.03
Extension		
aPT (Nm)	56.61 ± 14.69 *	66.02 ± 10.57
rPT (Nm/kg)	0.89 ± 0.26 *	1.11 ± 0.19
Flexion		
aPT (Nm)	35.01 ± 7.96	36.43 ± 7.00
rPT (Nm/kg)	0.54 ± 0.16 *	0.62 ± 0.15
H/Q	0.62 ± 0.15 *	0.55 ± 0.08

Note: KOA group is knee osteoarthritis group, CON group is control group, TMM is total muscle mass, LMM is lower limbs muscle mass, TFM is total fat mass, LFM is lower limbs fat mass, aPT is absolute peak torque, rPT is relative peak torque, and H/Q is the relative peak torque ratio of knee flexion and extension. * *p* < 0.05.

**Table 4:** Correlation between the relative peak torque of the knee joint and body composition of participants between the KOA and CON groups.

Group	rPT (Nm/kg)	Correlation Coefficient r			
		TFM (%)	TMM (%)	LFM (%)	LMM (%)
KOA group	Extension	−0.385 *	0.338	0.079	0.414 *
	Flexion	−0.503 *	0.462 *	0.100	0.532 *
CON group	Extension	−0.701 *	0.510 *	−0.490 *	0.478 *
	Flexion	−0.573 *	0.690 *	−0.539 *	0.624 *

Note: KOA group is knee osteoarthritis group, CON group is control group, TMM is total muscle mass, LMM is lower limbs muscle mass, TFM is total fat mass, LFM is lower limbs fat mass, and rPT is relative peak torque. * *p* < 0.05.
